# Clinical usefulness of library and information services in Japan: The detailed use and value of information in clinical settings

**DOI:** 10.1371/journal.pone.0199944

**Published:** 2018-06-28

**Authors:** Yukiko Sakai, Yoko Sato, Masae Sato, Makiko Watanabe

**Affiliations:** 1 School of Library and Information Science, Keio University, Tokyo, Japan; 2 The Value Study Working Group, The Japan Medical Library Association, Tokyo, Japan; 3 Division of Biomedical Engineering, National Defense Research Institute, National Defense Medical College, Tokorozawa, Saitama, Japan; 4 Chibaken Saiseikai Narashino Hospital, Narashino, Chiba, Japan; 5 Clinical Research Institute, Kanagawa Children’s Medical Center, Yokohama, Kanagawa, Japan; Medical University Graz, AUSTRIA

## Abstract

**Objectives:**

Considering that there is a lack of evidence regarding the contribution of library and information services to evidence-based medicine in actual clinical practice in Japan, the purpose of the study is to explore the current status of use and value of library and information services in clinical settings to examine the usefulness of information in implementing evidence-based medicine (EBM) into practice.

**Methods:**

A Web-based survey was conducted at seven sites (hospitals with 300–1,200 beds) and interviews conducted at five sites to investigate information behavior among health professionals (physicians, residents, and nurses) in 2016, replicating the Value Study carried out in the United States in 2010 and 2011. Using a critical incident technique, respondents answered questions about their information topics, information resources used, search location, access points, and evaluation of the information.

**Results:**

Analysis from 598 valid responses (275 physicians, 55 residents, and 268 nurses) revealed the characteristics of information use and recognition of the value of information. Physicians and residents showed their information needs regarding clinical care using PubMed (80.4%, 65.5%), Ichushi-Web (61.8%, 63.6%), and UpToDate (40.4%, 65.5%). While physicians rely more on electronic journals (37.8%), residents use more hybrid resources including Japanese print books (38.2%) and online books (30.9% for Japanese, 32.7% for English) to confirm their knowledge. Nurses need more information close to patients and explore a wider variety of information resources such as Japanese print books (60.4%), Ichushi -Web (40.3%), Japanese online books (20.5%), and websites of academic organizations (19.0%). Although the overall recognition of the value of information was relatively modest, concrete changes in clinical practice were found in some areas. Environments with insufficient information and availability of electronic resources should be improved to increase the use of library and information services for implementing EBM.

## Introduction

### Library and information services for health professionals in Japan

Systematic library and information services for health professionals in Japan are provided mainly by medical and hospital libraries. While in accordance with the Standards for Establishment of Universities [1] medical libraries are required at the 82 academic medical centers (Note: 81 at the time of the survey), only 548 out of the 8,429 hospitals have met this obligation and established libraries as the regional Medical Care Support Hospitals in accordance with the Medical Care Act [2]. Compared to academic medical libraries, which hold 198,870 volumes, subscribe to 9,271 print and 11,574 electronic journals, and are served by 9.2 staff members on average, hospital libraries are much smaller. An average hospital library has 3,950 books and subscribes to 87 print and 21.5 electronic journals serving users at a hospital with 450 beds with one librarian. (Note: this is the average values for 67 member academic libraries of The Japan Medical Library Association for the fiscal year 2016 and 102 member hospital libraries of the Japan Hospital Library Association for the fiscal year 2015).

### Evidence-based medicine in Japan

Evidence-Based Medicine (EBM) was introduced in the early 1990s in Japan, and the Ministry of Health has been encouraging EBM practice, especially supporting activities to develop clinical guidelines, since the late 1990s [3].

After the implementation of EBM and diffusion of clinical guidelines in Japan, there has been scant research on information needs and use among health professionals [4]. Evidence about how library and information services have contributed to EBM except in expert searching aimed at developing clinical guidelines is not known, even though Evidence-Based Librarianship (EBL)/ Evidence-Based Library and Information Practice (EBLIP) was concurrently proposed for medical and hospital librarians [5].

## Literature review

### Literature on the use and value of information in clinical settings in English-speaking countries

Research on clinical usefulness of library and information services has been widely conducted since the 1970s in English-speaking countries. A literature review prior to the Value Study in the United States listed 111 studies after 1975 showing the growth (e.g., 10 from 1975–1984, 23 from 1985–1994, 55 from 1995–2004) and stating the strength of evidence in terms of appropriate research designs impacted by EBM [6]. Another systematic review, which measures effects of librarian-provided services in health-care settings, in comparison, analyzes only 25 articles selected with rigorous criteria. The articles between 1986 and 2013 report 22 studies conducted in five countries including 12 randomized control trials [7].

The largest attempt to measure the impact of library and information services in clinical settings is the Value Study in the United States. The multi-centered study was conducted in 2010 and 2011, collecting 16,122 responses from health professionals working in 118 healthcare facilities in the United States and Canada. The overview article [8] reported that 3/4 of the respondents handled aspects of the patient care differently as a result of the information, including positive changes and avoidance of negative events. In addition to evaluation of the information, the study revealed detailed information use in clinical settings including information topics, search location, and access points.

The Value Study in the United States has been followed by three secondary analyses so far. The focal analyses concluded that the electronic collections and services provided by libraries contributed to patient care quality [9]. Another analysis focusing on nurses’ information use found that more information use and asking librarians for help resulted in positive clinical outcomes [10]. The latest follow-up analysis also showed that increased information use was related to more positive changes and avoidance of negative events [11].

### Literature on the use and value of information in clinical settings in Japan

The only study pursuing the direct effect of information in clinical settings in Japan is a survey conducted at 151 hospitals collecting 585 responses from residents in 2015 [12]. The purpose of the study was to reveal use of clinical guidelines in clinical settings and obstructive factors. They investigated IT environments at hospitals, information behavior among residents, and Quality Indicators (QI) as clinical outcomes. They found that IT environments were related to QI due to residents’ preference for electronic information resources.

A comprehensive survey on general information use and needs among physicians was conducted in 2000, during the early stages of EBM as well as the Internet in Japan, which collected 949 responses [13]. The respondents used information for clinical practice, being up-to-date on the latest knowledge in their own field, obtaining knowledge in other fields as well as to provide explanations to patients. The obstacles to obtaining information were unavailability of appropriate information, too much time, and too much cost.

As result of the literature review, it is clear that Japanese studies do not show the detailed use and the direct effect of information among various health professionals for clinical practice as opposed to studies in English-speaking countries. The purpose of this study is to explore the current status of use of library and information services in clinical settings to examine the clinical usefulness of information to implementing EBM practice based on the EBL/EBLIP approach, replicating the Value Study in the United States [8].

## Methods

To clarify the current status of use and the value of information in clinical settings in Japan, a multi-centered survey was designed by The JMLA Value Study Working Group based on the protocol of the Value Study in the United States. The questionnaire in the “Facilitator Handbook” in Appendix A [8] was translated into Japanese with some modification, for example, the addition of Japanese information resources as selections of the resource question and the deletion of the question to let respondents write the actual time saved as a result of the information for an appropriate and easy answer among Japanese respondents.

The survey questions consist of two large sections (i.e., information behavior in clinical settings and respondents’ profiles; see S1 Appendix and S2 Appendix). The information behavior section involves questions about one specific instance of patient care in the last six months that caused information seeking using a critical incident technique. The questions include those about the diagnosis of the patient, information topics, information resources, search location, access and use points, success of the information seeking, evaluation of information resources, evaluation of information, positive changes caused by the information, negative events avoided by the information, and evaluation of library and other information sources.

After obtaining ethical approval from the Institutional Review Board of Faculty of Letters, Keio University, the Working Group constructed the web-based survey site using the Realtime Evaluation Assistance System (REAS) provided publicly by the Open University of Japan for research and education purposes. Participating sites were recruited through public relations tools of JMLA. The survey was undertaken at seven sites (two in the first phase and five in the second phase) and interviews at five sites (two and three, respectively) to discover more detailed and general information behavior in cooperation with librarians working at the sites in 2016. Although the invitation to the study was open to academic medical centers and hospitals, all the sites are hospitals with about 300–1200 beds.

Chi-squared tests were used to compare categorical variables. When significant bias was observed on the chi-squared test (p < 0.05), a residual analysis was performed to determine which cell numbers in the cross-table represented sources of bias (p < 0.05). In the analysis of continuous values, a Mann–Whitney U Test was used for comparison between the two groups, and a Steel–Dwass test was used for the multiple comparisons among three groups. The influences of the number of used information resources on the results of the information searching behavior were analyzed by adjusting for demographic factors such as professional group, age, years of experience, and sex. When the dependent variable was a binary variable, multivariate logistic regression analyses were performed, and the odds ratio (OR), 95% CI, and p-value were calculated using the Wald test. When the dependent variable was continuous value, multiple regression analyses were performed, and the multiple regression coefficient, 95% CI, and p-value were calculated using the least-squares method. All analyses were performed using R version 3.3.2 using the “chisq.test”, “lm”, and “glm” functions (The R Foundation for Statistical Computing, Vienna, Austria).

## Results

### Respondents

**Fig 1:**
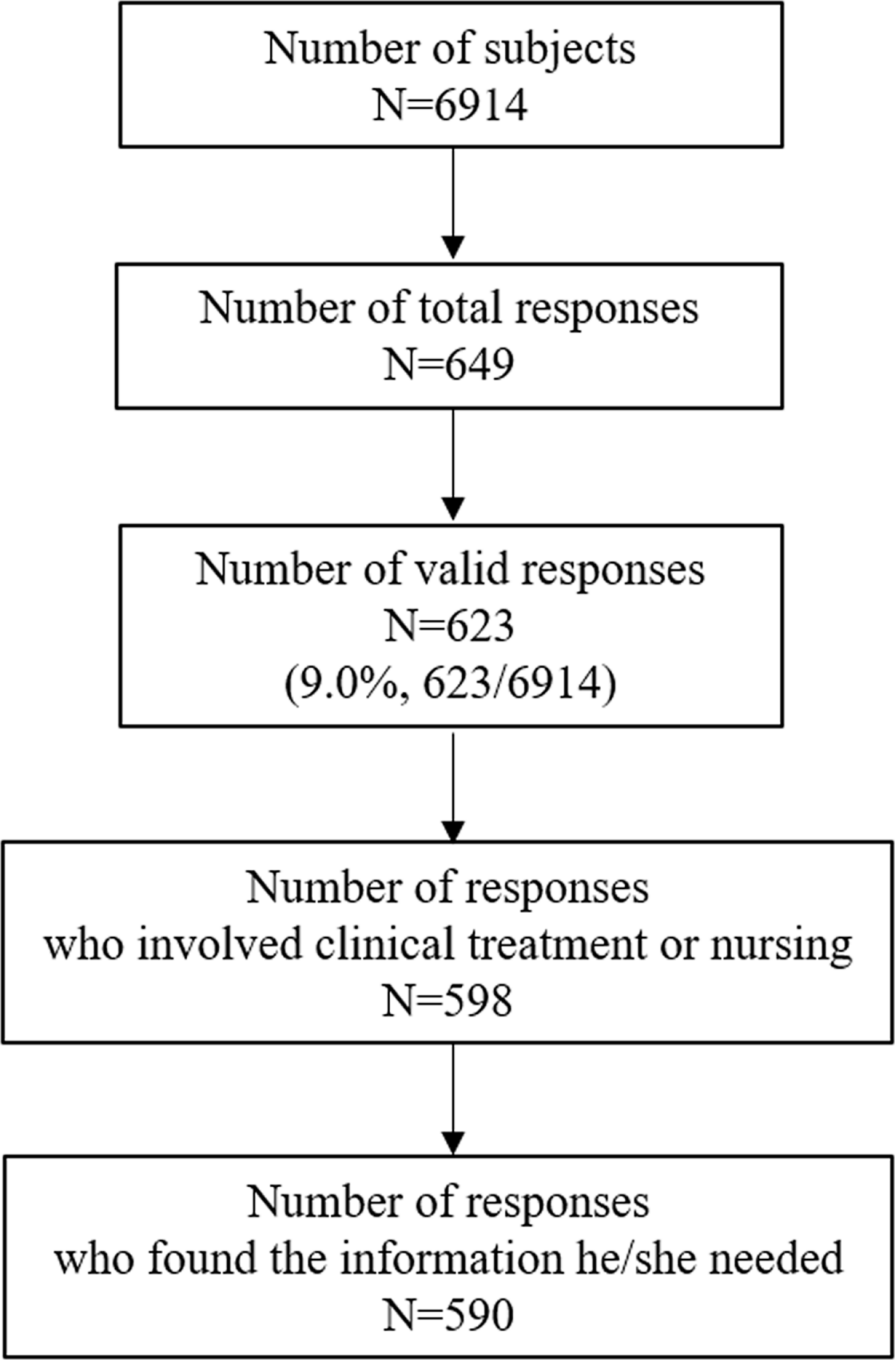
Follow up on responses.

The number of valid responses was 623, giving a response rate of 9.0% (Fig 1). Analysis was done for 623 responses, 598 responses from those who were involved in clinical treatment or nursing, and 590 responses from those who found the information he/she needed.

The response rate varied from 4.0% to 12.7%, depending on the participating sites. Moreover, the response rate by professional groups was 21.9% (282/1,289) for physicians; 11.9% (55/464) for residents; 5.4% (278/5,161) for nurses. The breakdown of professional groups was 45.3% (282/623) for physicians, 8.8% (55/623) for residents, and 44.6% (278/623) for nurses (Table 1). The overall ratio of men to women was 0.84 (285/338); the medical field is male dominated as evidenced by the following men-to-women ratios: 3.70 (222/60) for physicians or 2.24 (38/17) for residents, while 0.09 (24/254) for nurses (Table 1). The major age group was 30–39 (32.3%, 201/623) overall and 30–39 for physicians (37.9%, 107/282), while it was 20–29 for residents (67.3%, 37/55) and for nurses (41.7%, 116/278) (S1 Table). The number of years of experience as healthcare professionals varied from less than two years (12.2%, 76/623) to more than 20 years (26.8%, 167/623)(S2 Table). The major group varied according to professional group: 10–15 years for physicians (24.1%, 68/282); less than two years and/or 2–5 years for residents (45.5% each, 25/55); and more than 20 years for nurses (21.9%, 61/278) (S2 Table). Of a total of 27 interviewees, there were 9 physicians, 7 residents, and 11 nurses.

**Table 1:** Breakdown of responses.

Professional groups	N	(%)	Men/Women
Physicians	282	(45.3%)	3.70
Residents	55	(8.8%)	2.24
Nurses	278	(44.6%)	0.09
Others	8	(1.3%)	0.14
Total	623	(100.0%)	0.84

### Information-searching behavior

A total of 598 participants (46.0%, 275 physicians; 9.2%, 55 residents; 44.8%, 268 nurses), who were involved in clinical treatment or nursing, were asked about their information seeking for a specific patient care case in the last six months. Diagnoses of the patient are shown in the S3 Table consisting of two tables due to the different analyses for the first and second phase. The most frequent diagnosis in the first phase, in which respondents chose multiple selections, was “infectious diseases and parasitic diseases” (28.5%, 67/235) followed by “diseases of cardiovascular system” (21.3%, 50/235). In the second phase, in which respondents chose only one selection, most respondents in all the professional groups sought information for patients who suffered from “cancer” (32.0%, 116/363).

The questions about the type of information, information resources, searching locations, and access points were asked.

#### Type of information

The most needed type of information was “therapy” among all professional groups (70.2% for physicians, 67.3% for residents, and 60.4% for nurses) as shown by the yellow-tinted values in Table 2. The second most was “diagnosis” among physicians and residents (56.4%, 58.2%), while “drug information” for nurses (44.4%) as shown in the green-tinted values. “Information for patient” and “adverse effects” made the top five only among nurses. There was a difference in the number of people who needed “information for patient” among professional groups (S4 Table, p<0.001), and nurses needed “information for patients” more frequently (p<0.05). On the other hand, there was no difference among professional groups in “adverse effects” (p = 0.333).

**Table 2:** Type of information needed to answer the question among each professional group.

			Professional groups					
	Overall		Physicians		Residents		Nurses	
	(N = 598)		(N = 275)		(N = 55)		(N = 268)	
Type of information	N	%*	N	%*	N	%*	N	%*
Therapy information	392	65.6%	193	70.2%	37	67.3%	162	60.4%
Drug information	263	44.0%	123	44.7%	21	38.2%	119	44.4%
Diagnosis	248	41.5%	155	56.4%	32	58.2%	61	22.8%
Prognosis (outcome)	188	31.4%	123	44.7%	19	34.5%	46	17.2%
Clinical guidelines	171	28.6%	102	37.1%	18	32.7%	51	19.0%
Clinical procedure	151	25.3%	90	32.7%	15	27.3%	46	17.2%
Adverse effects	144	24.1%	66	24.0%	9	16.4%	69	25.7%
Information for patient	101	16.9%	21	7.6%	4	7.3%	76	28.4%
Patient safety	68	11.4%	30	10.9%	2	3.6%	36	13.4%
Other	38	6.4%	8	2.9%	0	0.0%	30	11.2%

Respondents were able to select all that applied. The top five items are colored in yellow (first), green (second), blue (third), purple (fourth), and pink (fifth).

* The denominator of the percentage is the number of each group.

#### Information resources

The most used information resource among physicians and residents was PubMed (Table 3, 80.4%, 65.5%). Residents used UpToDate (65.5%) as well at the same rate. In comparison, nurses used Japanese books in print (60.4%) the most.

**Table 3:** Resources used to search for the information needed to answer the question.

				Professional groups					
		Overall		Physicians		Residents		Nurses	
		(N = 598)		(N = 275)		(N = 55)		(N = 268)	
Information resource		N	%*	N	%*	N	%*	N	%*
(J)	Ichushi Web^a^	313	52.3%	170	61.8%	35	63.6%	108	40.3%
(J)	Books (print)	279	46.7%	96	34.9%	21	38.2%	162	60.4%
(E)	PubMed	276	46.2%	221	80.4%	36	65.5%	19	7.1%
(E)	UpToDate	151	25.3%	111	40.4%	36	65.5%	4	1.5%
(E)	Books (print)	125	20.9%	72	26.2%	13	23.6%	40	14.9%
(E)	Electronic journals	125	20.9%	104	37.8%	9	16.4%	12	4.5%
(J)	Web site of academic organizations	125	20.9%	65	23.6%	9	16.4%	51	19.0%
(J)	Books (online)	116	19.4%	44	16.0%	17	30.9%	55	20.5%
(E)	Books (online)	99	16.6%	62	22.5%	18	32.7%	19	7.1%
(J)	Printed magazines	95	15.9%	50	18.2%	8	14.5%	37	13.8%
(J)	Electronic journals	94	15.7%	64	23.3%	9	16.4%	21	7.8%
(E)	Web site of academic organizations	73	12.2%	46	16.7%	5	9.1%	22	8.2%
(E)	Printed magazines	51	8.5%	35	12.7%	8	14.5%	8	3.0%
(E)	ClinicalKey (Elsevier)	48	8.0%	36	13.1%	12	21.8%	0	0.0%
(E)	Cochrane Library	35	5.9%	30	10.9%	1	1.8%	4	1.5%
Not sure		34	5.7%	2	0.7%	0	0.0%	32	11.9%
(J)	Medical Information Network Distribution Service^b^	32	5.4%	15	5.5%	4	7.3%	13	4.9%
(E)	Clinical Evidence (BMJ)	30	5.0%	22	8.0%	7	12.7%	1	0.4%
(J)	Other	27	4.5%	5	1.8%	1	1.8%	21	7.8%
(J)	Current Index to Japanese Nursing Literature^c^	24	4.0%	0	0.0%	0	0.0%	24	9.0%
(E)	DynaMed	22	3.7%	15	5.5%	5	9.1%	2	0.7%
(J)	JDream III	16	2.7%	6	2.2%	0	0.0%	10	3.7%
(E)	Other	13	2.2%	3	1.1%	0	0.0%	10	3.7%
(E)	CINAHL	6	1.0%	0	0.0%	1	1.8%	5	1.9%
Resources except for Englinsh and Japanse		3	0.5%	3	1.1%	0	0.0%	0	0.0%

Respondents were able to select all that applied. Please note that not all the resources are available at survey sites. For example, UpToDate is available only at five out of seven survey sites.

The top five items are colored in yellow (first), green (second), blue (third), purple (fourth), and pink (fifth). (J) is a Japanese resource, and (E) is an English resource.

* The denominator of the percentage is the number of each group.

^a^ Japanese medical bibliographic database: http://www.jamas.or.jp/about/english.html.

^b^ https://minds.jcqhc.or.jp/english/english.php

^c^ http://www.nurse.or.jp/nursing/education/library/sakuin.html

There were differences in the number of people who used PubMed and UpToDate among professional groups (S5 Table, both p<0.001), and physicians and residents used them most frequently (both p<0.05). There was a difference in the number of people who used Japanese books in print among professional groups (p<0.001), and nurses used these was most frequently (p<0.05).

The median number of used information resources was 4 (interquartile range [IQR]:2–7) for physicians, 4 (IQR:3–7) for residents, and 2 (IQR:1–4) for nurses. The difference was not shown between physicians and residents (p = 0.936), while it was shown between physicians and nurses (p<0.001) and between residents and nurses (p<0.001).

#### Physical location in which you conducted or requested your search for information

The physicians and residents conducted or requested searches for information mostly at their offices (Table 4, 85.5%, 87.3%), while the nurses most often searched in the library (64.9%). There were differences among professional groups in the number of people who searched at their offices and patient care units (S6 Table, both p<0.001), and physicians and residents more frequently searched at their offices and patient care unit (both p<0.05). Furthermore, there was a difference in the number of people who searched at the library among professional groups (p<0.001); nurses more frequently searched at the library (p<0.05).

**Table 4:** Physical location you conducted or requested your search for information.

			Professional groups					
	Overall		Physicians		Residents		Nurses	
	(N = 598)		(N = 275)		(N = 55)		(N = 268)	
Location	N	%*	N	%*	N	%*	N	%*
Office	386	64.5%	235	85.5%	48	87.3%	103	38.4%
In the library (the physical place)	309	51.7%	108	39.3%	27	49.1%	174	64.9%
Home	191	31.9%	92	33.5%	18	32.7%	81	30.2%
Patient care unit	69	11.5%	54	19.6%	9	16.4%	6	2.2%
Other	22	3.7%	2	0.7%	0	0.0%	20	7.5%

Respondents were able to select all that applied. The top five items are colored in yellow (first), green (second), blue (third), purple (fourth), and pink (fifth).

* The denominator of the percentage is the number of each group.

#### Access to the information resource used

Physicians and residents accessed to the information resource mostly on their institution’s library web site (Table 5, 71.3%, 78.2%), while nurses accessed it in their institution’s library (59.3%). There were differences among professional groups in the number of people who accessed information on their institution’s library website and in their institution’s library (S7 Table, both p<0.001). Physicians and residents accessed the information on their institution’s library website more frequently, while nurses accessed the information in their institution’s library more frequently (both p<0.05).

**Table 5:** Access to the information resource used.

			Professional groups					
	Overall		Physicians		Residents		Nurses	
	(N = 598)		(N = 275)		(N = 55)		(N = 268)	
Access point	N	%*	N	%*	N	%*	N	%*
On your institution's library website	336	56.2%	196	71.3%	43	78.2%	97	36.2%
In your institution's library	281	47.0%	99	36.0%	23	41.8%	159	59.3%
Search engine such as Goggle	252	42.1%	128	46.5%	20	36.4%	104	38.8%
Personal/departmental subscription	80	13.4%	41	14.9%	7	12.7%	32	11.9%
Asked your librarian or library staff	72	12.0%	40	14.5%	7	12.7%	25	9.3%
Bookmarked website	47	7.9%	29	10.5%	6	10.9%	12	4.5%
On other institution's library website	41	6.9%	22	8.0%	2	3.6%	17	6.3%
Asked medical representative of pharmaceutical company	23	3.8%	17	6.2%	3	5.5%	3	1.1%
Not sure	14	2.3%	0	0.0%	0	0.0%	14	5.2%
In other institution's library	13	2.2%	3	1.1%	0	0.0%	10	3.7%
Nothing	11	1.8%	3	1.1%	0	0.0%	8	3.0%
Asked colleague	9	1.5%	5	1.8%	1	1.8%	3	1.1%
Other	9	1.5%	4	1.5%	0	0.0%	5	1.9%
Asked other librarian or library staff	1	0.2%	1	0.4%	0	0.0%	0	0.0%

Respondents were able to select all that applied. The top five items are colored in yellow (first), green (second), blue (third), purple (fourth), and pink (fifth).

* The denominator of the percentage is the number of each group.

### Value of the information

As a result of information seeking, 98.7% (590/598) of respondents said they found (i.e., “completely found” or “partially found”) information they needed. The breakdown of professional groups showed that 99.6% (274/275) of physicians, 100% (55/55) of residents, and 97.8% (261/267) of nurses found the information they sought. There was a difference among professional groups in whether they found what they were looking for or not (p = 0.049), with more doctors finding the information sought, while more nurses stated they “did not” find the information (both p<0.05).

The median number of information resources used by respondents who found the information they needed was 3 (IQR:2–5), while the number of resources used by respondents who did not find the information was 1 (IQR:1–2.25). In the analysis of the association between the number of used information resources and whether or not participants found the information, by adjusting for demographic factors, we did not find an association (odds ratio [OR]: 1.95, 95% confidence interval (CI): 0.93–4.10, p = 0.078).

#### Evaluation of the information obtained

Respondents numbering 590 (46.5%, 274 physicians; 9.3%, 55 residents; 44.2%, 261 nurses), who found the information they needed, answered the questions about the evaluation of the information, positive changes, avoiding negative events, and the importance of information sourced including library and information services.

Most physicians and residents evaluated the information they obtained as “information was relevant” (Table 6, 96.4%, 98.2%), while most nurses said “the information provided new knowledge” (88.9%). There were differences in the number of people who thought “the information was relevant” and “the information provided new knowledge" among professional groups (S8 Table, p<0.001, 0.022). More physicians and residents thought “the information was relevant” and “the information provided new knowledge” (both p<0.05). Most of the respondents who agreed with statements, “the information was relevant,” “the information refreshed my memory of detail or facts,” “the information substantiated my prior knowledge or belief,” “the information was of clinical value,” and “the information resulted in a better informed clinical decision,” used more information resources than respondents who did not agree with these statements (S9 Table, all p<0.037).

**Table 6:** Agreement with statements about the information used.

				Professional groups					
		Overall (N = 590)		Physicians (N = 274)		Residents (N = 55)		Nurses (N = 261)	
	Category	N	%*	N	%*	N	%*	N	%*
The information provided new knowledge.	Yes	545	(92.4%)	260	(94.9%)	53	(96.4%)	232	(88.9%)
The information was relevant.	Yes	509	(86.3%)	264	(96.4%)	54	(98.2%)	191	(73.2%)
The information will be of use in the future.	Yes	492	(83.4%)	248	(90.5%)	52	(94.5%)	192	(73.6%)
The information was of clinical value.	Yes	468	(79.3%)	253	(92.3%)	50	(90.9%)	165	(63.2%)
The information substantiated my prior knowledge or belief.	Yes	441	(74.7%)	210	(76.6%)	46	(83.6%)	185	(70.9%)
The information was accurate.	Yes	437	(74.1%)	213	(77.7%)	49	(89.1%)	175	(67.0%)
The information resulted in a better informed clinical decision.	Yes	411	(69.7%)	228	(83.2%)	45	(81.8%)	138	(52.9%)
The information contributed to higher quality of care.	Yes	381	(64.6%)	214	(78.1%)	43	(78.2%)	124	(47.5%)
The information refreshed my memory of detail or facts.	Yes	322	(54.6%)	162	(59.1%)	30	(54.5%)	130	(49.8%)
The information was current.	Yes	310	(52.5%)	183	(66.8%)	34	(61.8%)	93	(35.6%)
Having information saved me time.	Yes	263	(44.6%)	146	(53.3%)	27	(49.1%)	90	(34.5%)

The top five items are colored in yellow (first), green (second), blue (third), purple (fourth), and pink (fifth).

* The denominator of the percentage is the number of each group.

#### Any changes as a result of the information

The percentage of respondents who recognized any changes as a result of the information was 75.1% (443/590). Fewer nurses (63.2%, 165/261) felt the changes compared to physicians (84.3%, 231/274) and residents (85.5%, 47/55) (p<0.05).

Respondents who recognized the changes used a median of 3 (IQR:2–6) information resources, and respondents who did not recognize the changes used a median of 2 (IQR:1–4) (p<0.001).

#### Positive changes as a result of the information

The percentage of respondents who recognized positive changes as a result of the information was 88.6% (523/590). Fewer nurses 82.4% (215/261) felt the positive changes compared to physicians (94.2%, 258/274) and residents (90.9%, 50/55) (p<0.05).

Respondents who recognized the positive changes used a median of 3 (IQR:2–6) information resources, and respondents who did not recognize the changes used a median of 2 (IQR:1–4) (p<0.001). The difference was supported by multivariate analysis adjusted for demographic factors (OR: 1.19, 95%, CI: 1.03–1.36, p = 0.015).

The 523 respondents who recognized positive changes were asked about the type of changes. Physicians and residents most frequently indicated “choice of test” (Table 7, 73.3%, 74.0%), while nurses more frequently chose “changed advice given to patient” (62.3%). There were differences in the number of people who chose “choice of test” and “changed advice given to patient” as a positive change among professional groups (S10 Table, p<0.001). Physicians and residents chose “choice of test” most frequently, and nurses chose “changed advice given to patient” most frequently (both p<0.05).

**Table 7:** Positive changes as a result of the information.

				Professional groups					
		Overall (N = 523)		Physicians (N = 258)		Residents (N = 50)		Nurses (N = 215)	
Changes reported	Category	N	%*	N	%*	N	%*	N	%*
Choice of test	Yes	254	(48.6%)	189	(73.3%)	37	(74.0%)	28	(13.0%)
Diagnosis	Yes	213	(40.7%)	163	(63.2%)	33	(66.0%)	17	(7.9%)
Changed advice given to patient	Yes	200	(38.2%)	59	(22.9%)	7	(14.0%)	134	(62.3%)
Choice of drugs	Yes	145	(27.7%)	98	(38.0%)	18	(36.0%)	29	(13.5%)
Choice of treatments	Yes	109	(20.8%)	84	(32.6%)	15	(30.0%)	10	(4.7%)
Post-hospital care or treatment	Yes	103	(19.7%)	18	(7.0%)	4	(8.0%)	81	(37.7%)
Other	Yes	41	(7.8%)	6	(2.3%)	0	(0.0%)	35	(16.3%)
Length of stay (reduced)	Yes	21	(4.0%)	14	(5.4%)	4	(8.0%)	3	(1.4%)

Respondents were able to select all that applied. The top five items are colored in yellow (first), green (second), blue (third), purple (fourth), and pink (fifth).

* The denominator of the percentage is the number of each group.

The median number of positive changes was 2 (IQR: 1–3) for physicians, 2 (IQR: 1–3) for residents, and 1 (IQR: 1–2) for nurses in descending order. The difference was shown between physicians and nurses (p<0.001) and between residents and nurses (p<0.001). Furthermore, the number of positive changes was positively related to the number of information resources used (β Coefficient: 0.33, 95%, CI: 0.25–0.41, p<0.001).

#### Avoided unwelcome events as a result of the information

The percentage of respondents who recognized that they avoided unwelcome events as a result of the information was 53.7% (317/590). Fewer nurses 46.7% (122/261) felt the impact of information on negative events compared to physicians 58.8 (161/274) and residents 61.8% (34/55) (p<0.05).

Respondents who recognized the avoided unwelcome events used a median of 4 (IQR: 2–6) information resources, and respondents who did not recognize the events used a median of 3 (IQR:1–4). The difference was supported by multivariate analysis adjusted for demographic factors (OR: 1.12, 95%, CI: 1.05–1.26, p = 0.001).

The 317 respondents who recognized the avoided unwelcome events were asked about the type of events. Physicians and residents most frequently chose “additional tests and procedures” (Table 8, 51.6%, 61.8%), while nurses most often chose “patient misunderstanding of disease” (39.3%). There were differences among professional groups in the number of people who chose “additional tests and procedures” and “patient misunderstanding of disease” as an avoided adverse effect (S11 Table, p<0.001). Physicians and residents chose “additional tests and procedures” as an avoided adverse effect more frequently, and nurses chose “patient misunderstanding of disease” more frequently (both p<0.05).

**Table 8:** Events avoided as a result of the information.

				Professional groups					
		Overall (N = 317)		Physicians (N = 161)		Residents (N = 34)		Nurses (N = 122)	
Adverse event avoided	Category	N	%*	N	%*	N	%*	N	%*
Additional tests or procedures	Yes	118	(37.2%)	83	(51.6%)	21	(61.8%)	14	(11.5%)
Adverse drug reaction or interaction	Yes	107	(33.8%)	59	(36.6%)	12	(35.3%)	36	(29.5%)
Patient misunderstanding of disease	Yes	82	(25.9%)	30	(18.6%)	4	(11.8%)	48	(39.3%)
Other	Yes	38	(12.0%)	10	(6.2%)	2	(5.9%)	26	(21.3%)
Misdiagnosis	Yes	25	(7.9%)	23	(14.3%)	2	(5.9%)	0	(0.0%)
Hospital admission	Yes	22	(6.9%)	11	(6.8%)	4	(11.8%)	7	(5.7%)
Surgery	Yes	18	(5.7%)	15	(9.3%)	3	(8.8%)	0	(0.0%)
Patient mortality	Yes	17	(5.4%)	9	(5.6%)	5	(14.7%)	3	(2.5%)
Language/culture misunderstanding	Yes	17	(5.4%)	4	(2.5%)	0	(0.0%)	13	(10.7%)
Hospital acquired infection	Yes	14	(4.4%)	3	(1.9%)	1	(2.9%)	10	(8.2%)
Hospital readmission	Yes	10	(3.2%)	3	(1.9%)	2	(5.9%)	5	(4.1%)
Medication error	Yes	9	(2.8%)	6	(3.7%)	2	(5.9%)	1	(0.8%)
Regulatory non-compliance	Yes	8	(2.5%)	3	(1.9%)	0	(0.0%)	5	(4.1%)

Respondents were able to select all that applied. The top five items are colored in yellow (first), green (second), blue (third), purple (fourth), and pink (fifth).

* The denominator of the percentage is the number of each group.

The median number of unwelcome events was 1(IQR: 0–2) for residents, 1 (IQR: 0–1) for physicians, and 0 (IQR:1–0) for nurses in descending order. The difference was shown between physicians and nurses (p = 0.002) and between residents and nurses (p = 0.025). Furthermore, the number of avoided unwelcome events was positively related to the number of information resources used (β Coefficient: 0.35, 95%, CI: 0.26–0.43, p<0.001).

#### Importance of the information received from different sources in relation to this medical situation

Physicians and nurses most often chose “library information resources” at (Table 9, 81.0%, 76.2%) as the important source among four kinds of clinical information sources, while residents most often chose “discussion with colleagues” (83.6%). There were differences in the number of people who rated “lab test” and “diagnostic imaging” as important among professional groups (S12 Table, both p<0.001). The number of physicians and residents who rated “lab test” and “diagnostic imaging” important were more frequent (both p<0.05).

**Table 9:** Importance of the information received from different sources in relation to this medical situation.

				Professional groups					
		Overall (N = 590)		Physicians (N = 274)		Residents (N = 55)		Nurses (N = 261)	
Source	Category**	N	%*	N	%*	N	%*	N	%*
Library information resources	Important	461	(78.1%)	222	(81.0%)	39	(70.9%)	200	(76.2%)
Discussion with colleagues	Important	447	(75.8%)	205	(74.8%)	46	(83.6%)	196	(74.7%)
Lab tests	Important	399	(67.6%)	205	(74.8%)	45	(81.8%)	149	(56.7%)
Diagnostic imaging	Important	383	(64.9%)	205	(74.8%)	44	(80.0%)	134	(51.0%)
Other	Important	61	(10.3%)	21	(7.7%)	7	(12.7%)	33	(12.3%)

Respondents were able to select all that applied. The top five items are colored in yellow (first), green (second), blue (third), purple (fourth), and pink (fifth).

* The denominator of the percentage is the number of each group.

** Category is changed 3 scales from 5 scales with the question about the importance of the information received from different sources. “Very important” and “Important” are categorized as “Important,” and “Not very important” and “Not important” are categorized as “Not Important.”

## Discussion

The web survey result is discussed with interview statements in terms of features of information behavior by professional groups (i.e., physicians, residents, and nurses) with international comparison, use of characteristic Japanese information resource, and recognition of the value of information in clinical settings in Japan.

### Features of information behavior by professional groups with international comparison

#### Physicians

Physicians in this study need more types of information because of their stronger needs direct to clinical practice such as therapy, diagnosis, and drug information as shown in the result of the U.S. Value Study (US-VS) collecting responses from health professionals in North America [8]. Compared to nurses, they need more information, especially about diagnosis, prognosis and outcome, and clinical guidelines, and they use more information resources.

The results on information resources showed a different tendency in comparison with the US-VS. Physicians use mainly PubMed, Ichushi-Web (Japanese medical bibliographic database), UpToDate, and electronic journals, which are all electronic. The preference for electronic resources in clinical settings is also supported by the result of another recent study in Japan [14]. However, the physicians in the current study use UpToDate (40.4%) and electronic journals (37.8%) a bit less than US-VS respondents (53.0%, 59.0%) and still more rely on print books (34.9% for Japanese, 26.2% for English) than those in North America (24.0%).

This modest reliance on electronic resources is thought to be due to less availability of electronic resources and/or their need for Japanese resources. For example, UpToDate was only available at five out of seven sites in this study and 49.0% (74/151) hospitals in another study in 2015[12]. A statement from an interviewee indicates both less availability of electronic journals in Japanese and their need for Japanese resources: “I use Ichushi-Web for searching, but full texts are hardly accessible.”

Physicians in this study conduct searches mostly from their offices and get to the resources through their institutions’ library web sites and search engines, similar to the respondents of the US-VS. However, they do search and access more in the institutions’ library (current 39.3%; US-VS 11.0%) (36.0%, 21.0%) and less at patient care units (19.6%, 34.0%). The reason for the difference may be caused by the limited information environment at patient care unit areas in Japanese hospitals as reported by Imanaka et al. [12] and reliance on print materials as shown in the results of the information resources section above.

#### Residents

Like physicians, residents also need types of information directed to clinical practice. The top two types, “therapy” and “diagnosis”, are also in common in the US-VS results. Residents use more information resources than nurses do.

Residents use mainly PubMed, UpToDate, and Ichushi-Web at the same level. The higher rate of using UpToDate among residents compared to that of physicians may indicate residents’ needs for the latest, most reliable medical knowledge related to EBM. A resident stated in the interview, “I usually follow and confirm clinical procedure that my attending doctor does, because I am still a first-year resident.”

Japanese books in print and electronic books both in English and Japanese are used by residents at some level (30.9–38.2%). This mixed use of print and electronic books corresponds the results of another study about electronic and print books among health professionals in 2016 [14], showing majority of hybrid use (86.7%). A resident in the current study also showed their different use of electronic and print books as “I use Year Note series [dictionary-like electronic books], but I prefer textbooks in print because I can easily write down a note.”

The interview revealed that residents frequently use their smartphones for quick searches. For example, residents said, “Using my iPhone, I usually use Google,” and, “I use my smartphone for a quick search when something comes to my mind at a hospital ward.”

#### Nurses

Nurses need information on therapy and drugs, followed by information for patients. Information for patients is needed more among nurses compared to physicians and residents.

The differences in types of information needed by nurses between the current study and the US-VS were shown by “information for patients” (28.4%, 44.0%) and “diagnosis” (22.8%, 45.0%). This lower profile in a specific area of clinical practice may be caused by the different range of responsibilities among Japanese nurses. The nursing standards in Japan define nursing practice under the initiative of medical doctors [15].

The main information resources for nurses are Japanese books in print and Ichushi-Web. Nurses in the US-VS, in contrast, use more electronic resources such as Micromedex (35.0%), electronic journals (30.0%), PubMed (25.0%), and electronic books (22.0%). The reason for reliance on print books in Japan is the unavailability of Japanese electronic materials. Considering of the fact that only 6.9% of electronic books are Japanese books held by academic libraries in Japan [16], the unavailability may not only an issue of library subscription but also of electronic publishing.

The preference for institution libraries as searching location and access points is caused by the reliance on print books as main information resources and limited information environment for nurses in hospitals. Nurses stated in the interview, “Only one computer is available at each office,” “Computers at the office are too few for nurses preparing for nursing research,” and “Computers for nurses are fewer than those for residents.” Therefore, fewer nurses can conduct searches at the office compared to physicians and residents. Moreover, very few conduct searches at patient units (2.2%). In contrast, nurses who participated in the US-VS indicated that they conduct searches at patient care unit more frequently (64.0%).

Using a search engine at the same level of US-VS (38.8%, 35.0%) seems related to the need for more practical information. A nurse said in the interview that nurses need nursing assessment sheets used by other hospitals for reference and that they developed their own sheet by adding some items to the sheet. As shown in another study’s exploration of the wide variety of information needs of nurses [17], they tend to rely on more books and use search engines as the threshold of information.

### Use of characteristic Japanese information resources

Ichushi-Web is an exclusive medical bibliographic database in Japan. This database is the second-most-used information resource among all the professional groups, probably due to the need for Japanese resources.

Clinical guidelines are considered as basic information resources for EBM. However, the guidelines are still modestly used information resources in Japan compared to North America as shown in the US-VS (current 37.1% and US-VS 54.0% for physicians; 32.7% and 59.0% for residents; 19.0% and 39.0% for nurses). There might be some issues for actual use of evidence in clinical guidelines, even though the guidelines have been developed after 2000s in Japan [3]. A couple of residents mentioned the obstacles and limitations in using clinical guidelines in the interview: “Because of the rapid development of drugs in hematology, I feel like I should look for the latest drug information by myself,” “We’ve been told that clinical guidelines fit only 80% of patients. Therefore, we should examine the original articles referred to in the guidelines to know whether we could follow the guidelines for our patient.”

The need for “information for patient” is less in the current study compared to that in the US-VS. Very few physicians and residents searched for “information for patient” (current 7.6%, 7.3%; US-VS 27.0%, 28.0%), while a slightly higher number of nurses searched for this information (28.4%, 44.0%).

### Recognition of the value of information in clinical settings in Japan

The current study found some level of recognition of the value of information in clinical settings, but some responses looked modest. For example, most of the respondents recognized either any changes or positive changes as a result of the information (75.1%, 88.6%) at the same level as the US-VS respondents (74.3%, 84.7%). The percentages of any changes in the current study and the US-VS were calculated as follows: 75.1% (443/590) and 74.3% (10,287/13,852). The numerators are the number of respondents who answered definitely or probably yes for the any change question (Question 1.7b) and the denominators are the number of respondents from the find question who valued the information completely or partially (Question 1.6). The percentages of positive changes were calculated differently due to the slightly different questions: 88.6%(523/590) for the current study and 84.7% (11,731/13,852) for the US-VS. For the current study, the numerator is the number of respondents who answered definitely or probably yes for the positive change question (Question 1.9) and the denominator is the number of respondents who valued the information completely or partially for the find question (Question 1.6). For the US-VS, the numerator is the number of respondents who did not select “not applicable” for the positive change question (Question 1.10, 13,852–2,121) and the denominator is the number of respondents who valued the information completely or partially for the find question (Question 1.6).

Moreover, some concrete changes were selected at even higher rate than that of the US-VS study. However, overall evaluation of the information in aspects of quality, cognitive value, contribution to quality of patient care, and time were relatively lower (44.6–92.4%) compared to US-VS ratings (85.0–99.0%). Considering the fact that the highest-rated item was “the information provided new knowledge” (92.4%), the majority of respondents in the current study might think of “information as knowledge” instead of “information as evidence for clinical practice.”

Higher or lower rates of selection of positive changes and avoided events could imply the characteristic clinical areas in Japan influenced by the information regardless of their recognition or evaluation of the information. Physicians and residents in the current study selected “diagnosis” (63.2%, 66.0%) and “choice of tests” (73.3%, 74.0%) rather than “choice of drugs” (38.0%, 36.0%) and “choice of treatment” (32.6%, 30.0%) as positive changes as a result of the information, while physicians (36.0%, 35%; 46.0%, 42.0%) and residents (42.0%, 40.0%; 52.0%, 43.0%) in the US-VS selected the other way. Given the fact that “additional test or procedures” was chosen as the most avoided event among physicians (current 51.6%; US 29.0%) and residents (61.8%, 32.0%), the effect of information was recognized more in the diagnostic phase.

“Changed advice given to patient” and “post-hospital care or treatment” were also selected as positive changes differently as compared to the US-VS. Nurses in the current study chose these change more frequently (current 62.3%, 37.7%; US 49.0%, 12.0%), although physicians (22.9%, 7.0%; 47.0%, 12.0%) and residents (14.0%, 8.0%; 45.0%, 15.0%) selected these change at a lower rate. The result may indicate the tendency of nurses’ roles involving working close to patients.

In regard to the number of information resources, the same tendency was shown in the results of the secondary analyses of the US-VS [10], [11]. More resources are related to higher evaluation of the information, more positive changes and avoided unwelcome events. This tendency may imply the empowerment among health professionals influenced by the information and the potential power of information contributing to EBM in actual clinical care.

## Conclusions

The current study revealed detailed use of information by physicians, residents, and nurses in clinical settings in Japan. Physicians and residents showed similar behavior, with some differences in electronic information resources. For example, residents sometimes perform quick searches using electronic resources on their smartphones, while confirming their basic medical knowledge in print books. They use UpToDate more than physicians do. Nurses showed different tendencies in aspects of topics, resources, search location, and access points due to the variety of their information needs and the availability of resources and the information environment. Characteristics of information behavior among professional groups is similar to the result of the US-VS, although some differences were shown, caused by a greater reliance on paper materials, differences in the information environment, and different ranges of job responsibilities.

Although respondents in the current study did less clearly recognize the value of the information overall, they actually noticed the concrete changes for their clinical practice. However, insufficient information environment and less availability of electronic resources in Japanese were implied in this study. This situation could be a matter of particular concern, because it has been considered as one of the most common barriers to EBM application [18]. As Imanaka et al. [12] pointed out, a more sufficient information environment as well as a higher availability of electronic resources may contribute to increased use of evidence in the actual clinical practice and enhance the recognition on the value of information.

The limitations of the current study involve two kinds of positive bias. First, participating sites were hospitals with relatively well-organized library services. They were recruited through librarians as facilitators of the study due to the community-based study design. This means that there was at least one librarian at each hospital. It was reported that there was no librarian at 55.5% of hospitals in Japan and only 26.7% offered library websites for convenient access to electronic resources [12]. Second, respondents might have been more active users of library and information services because the librarians, who acted as facilitators, invited them. In fact, all the nurse interviewees currently attend or did attend graduate schools and are considered as active library and information service users. Based on the biases, the result may not represent the situation in average hospitals in Japan.

Although this study collected a relatively small volume of data compared to the US-VS, there is no other data revealing detailed use of library-served information and its value in clinical settings, especially after introducing EBM in Japan. Due to the small number of respondents and possible biases mentioned above and the lack of data from academic medical centers, further studies collecting data including those from academic medical centers are required for better understanding of the clinical usefulness of library and information services in the EBL/EBLIP approach for contributing to EBM.

## Supporting information

S1 Appendix The survey questionnaire in Japanese. (PDF)

S2 Appendix The survey questionnaire in English. (PDF)

S1 File Aggregated data. (XLSX)

S2 File Subsettable dataset. (XLSX)

S1 Table Age category of all respondents. (XLSX)

S2 Table Years of experience of all respondents. (XLSX)

S3 Table Principal diagnosis of patients to whom your situation is related. Respondents in the first phase chose all that applied (multiple sections), and those in the second phase chose only one section. The top five items are colored in yellow (first), green (second), blue (third), purple (fourth), and pink (fifth).
(XLSX)

S4 Table The difference among professional groups of each type of information needed to answer the question. (XLSX)

S5 Table The difference among professional groups of resources used to search for the information needed to answer the question. (XLSX)

S6 Table The difference among professional groups of physical location they conducted or requested their search for information. (XLSX)

S7 Table The difference among professional groups of access to the information resource used. (XLSX)

S8 Table The difference among professional groups of agreement with statements about the information used. (XLSX)

S9 Table The association between the number of information resources used and statements about the information used. (XLSX)

S10 Table The difference among professional groups of positive changes as a result of the information. (XLSX)

S11 Table The difference among professional groups of events avoided as a result of the information. (XLSX)

S12 Table The difference among professional groups of the importance of the information received from different sources in relation to this medical situation. (XLSX)
