# Supplementary material for: Clinical usefulness of library and information services in Japan: The detailed use and value of information in clinical settings
Source: PLoS One. 2018 Jun 28;13(6):e0199944. doi: 10.1371/journal.pone.0199944 (PMC6023225; doi:10.1371/journal.pone.0199944)
Supplement: S1 Appendix — (PDF) [file pone.0199944.s001.pdf]

S1_File The survey questionnaire in Japanese *

「医療における情報のバリュー・スタディ」へようこそ！

○○○病院の医師、研修医、看護師のみなさま：
ご回答は、診療のために必要な図書館情報資源へのアクセスを評価し、改善するために役立ちます。依頼メールにもありました下記の「調査に関する情報」に同意いただき、回答を開始してください。

[調査に関する情報]
○[ラジオボタン]「調査に関する情報」に同意します

---

セクション 1：あなたの診療上の疑問を解決するための情報ニーズについてお尋ねします。

1.1. あなたのご職業は？
1. 医師・歯科医師
2. 研修医（前期）
3. 研修医（後期）
4. 看護師
5. 看護師（管理職）
6. その他(具体的に：　　　　　　　　　　　　　　)

1.1a. あなたの仕事は以下のうちどれですか？
1. 診療
2. 看護
3. 診療・看護
4. その他

1.1b あなたの仕事で当てはまるものを選んでください。（複数回答可）
1. 臨床研究
2. 基礎研究
3. 教育
4. 経営管理
5. その他（具体的に：　　　　　　　　　　　　　　）

[1.1a で 4 を選んだ方は、1.1b 回答終了後、2.1 に進んでください]

以降の質問については、最近 6 か月以内に、電子カルテや臨床検査結果等の医療情報以外に、診療上の疑問を解決するために各種データベースや電子ジャーナル等の図書館が扱うような情報資源を探したり使ったりした時のことを一つだけ思い出して、お答えください。

1.1c（第 1 期）患者の疾病や障害は何でしたか？（複数回答可）

1.1c（第 2 期）患者の疾病や障害は何でしたか？（1 つだけ選択）

1. 感染症および寄生虫症
2. がん・新生物
3. 血液及び造血器の疾患・免疫機構の障害
4. 内分泌、栄養および代謝疾患
5. 精神および行動の障害
6. 神経系の疾患
7. 眼および付属器の疾患
8. 耳および乳様突起の疾患
9. 循環器系の疾患
10. 呼吸器系の疾患
11. 消化器系の疾患
12. 皮膚及び皮下組織の疾患
13. 筋骨格系および結合組織の疾患
14. 腎尿路生殖器系の疾患
15. 妊娠、分娩および産じょく
16. 周産期に発生した病態
17. 先天奇形、変形および染色体異常
18. 損傷、中毒およびその他の外因による障害
19. その他（具体的に：　　　　　　　　　　　　）

引き続き、診療にあたって図書館情報資源を探したり使ったりした同じ時のことについてお答えください。

1.2. 疑問を解決するために、どのような種類の情報が必要でしたか？(複数回答可)

1. 薬物情報
2. 治療情報
3. 診療手技に関する情報
4. 診断に関する情報

5. 患者向け情報
6. 予後あるいはアウトカムに関する情報
7. 副作用情報
8. 患者の安全に関する情報
9. 診療ガイドライン
10. その他(具体的に：　　　　　　　　　　　　　　)

---

引き続き、診療にあたって図書館情報資源を探したり使ったりした同じ時のことについてお答えください。

1.3 必要な情報を探すために、どのような情報資源を使いましたか？(複数回答可)

英語の情報資源

1. 電子書籍
2. 本
3. Clinical Evidence（BMJ）
4. DynaMed
5. UpToDate
6. Cochrane Library
7. ClinicalKey (Elservier)
8. PubMed
9. CINAHL
10. 電子ジャーナル
11. 印刷物の雑誌
12. 学会 Web サイト
13. その他（具体的に：　　　　　　　　　　　　　　）

日本語の情報資源

14. 電子書籍
15. 本
16. 医中誌 Web
17. JDream III
18. 最新看護索引 Web
19. 電子ジャーナル
20. 印刷物の雑誌
21. 学会 Web サイト
22. Minds 医療情報サービス
23. その他（具体的に：　　　　　　　　　　　　　　）

24. 英語・日本語以外の情報資源（具体的に：　　　　　　　　　　　　　　　　）
25. わからない

---

引き続き、診療にあたって図書館情報資源を探したり使ったりした同じ時のことについてお答えください。

1.4 上記の情報資源にどのようにアクセスしましたか？(複数回答可)

1. 院内の図書館・室に足を運んだ
2. 院内の図書館・室の Web サイトからアクセスした
3. 院内の図書館・室の担当者に依頼した
4. 院外の図書館・室に足を運んだ
5. 院外の図書館・室の Web サイトからアクセスした
6. 院外の図書館・室の担当者に依頼した
7. 同僚に依頼した
8. 個人,医局や病棟の購読誌
9. 製薬企業の情報担当者から入手した
10. Google のような検索エンジンからアクセスした
11. お気に入り登録の Web サイトを探した
12. 該当なし
13. その他（具体的に：　　　　　　　　　　　　）
14. わからない

1.5 上記の情報資源をどこで探したり使ったりしましたか？(複数回答可)

1. 院内の図書館・室
2. 医局や病棟
3. 自宅
4. 診察室
5. その他（具体的に：　　　　　　　　　　　　）

---

引き続き、診療にあたって図書館情報資源を探したり使ったりした同じ時のことについてお答えください。

1.6 診療上の疑問を解決するために必要な情報は見つけられましたか？

1. 十分見つけられた
2. 一部だけ見つけられた
3. まったく見つけられなかった

[1.6 で「2.一部だけ見つけられた」「3.まったく見つけられなかった」と回答した方は 1.6a へ、その他の方は 1.7 へお進みください]

1.6a なぜ必要な情報が十分見つけられなかったのでしょうか？(複数回答可)

1. 時間が足りなかった
2. 必要な情報が存在しなかった
3. 必要な情報を発見できなかった
4. その他(具体的に：　　　　　　　　　　　　　　)

[1.6 で「3 必要な情報を発見できなかった」と回答された方は、1.7-12 は飛ばしてください]

---

引き続き、診療にあたって図書館情報資源を探したり使ったりした同じ時のことについてお答えください。

1.7 どの情報資源に、診療上の疑問解決に関連した情報が含まれていましたか？(複数回答可)

英語の情報資源

1. 電子書籍
2. 本
3. Clinical Evidence（BMJ）
4. DynaMed
5. UpToDate
6. Cochrane Library
7. ClinicalKey (Elservier)
8. PubMed
9. CINAHL
10. 電子ジャーナル
11. 印刷物の雑誌
12. 学会 Web サイト
13. その他（具体的に：　　　　　　　　　　　　　　）

日本語の情報資源

14. 電子書籍
15. 本
16. 医中誌 Web
17. JDream III
18. 最新看護索引 Web
19. 電子ジャーナル

20. 印刷物の雑誌
21. 学会 Web サイト
22. Minds 医療情報サービス
23. その他（具体的に：　　　　　　　　　　　　　　　　）

24. 英語・日本語以外の情報資源（具体的に：　　　　　　　　　　　　　　　　　　　）
25. わからない

---

引き続き、診療にあたって図書館情報資源を探したり使ったりした同じ時のことについてお答えください。

1.7a あなたが入手した情報によって、診療において何らかの違いはありましたか？

1. はい
2. どちらかといえば「はい」
3. どちらかといえば「いいえ」
4. いいえ

1.8 あなたが使った情報について、あてはまるものを選択してください。

(1) 診療上の疑問に関連したものだった

1. はい
2. いいえ
3. わからない

(2) 正確だった

1. はい
2. いいえ
3. わからない

(3) 最新だった

1. はい
2. いいえ
3. わからない

(4) 細かなことや事実に関する私の記憶をよみがえらせてくれた

1. はい
2. いいえ
3. わからない

(5) 私のそれまでの知識や考えを裏付けてくれた

1. はい
2. いいえ
3. わからない

(6) 新しい知識を得ることができた

1. はい
2. いいえ
3. わからない

(7) 臨床的価値があった

1. はい
2. いいえ
3. わからない

(8) より良い臨床上の決定につながった

1. はい
2. いいえ
3. わからない

(9) より質の高い診療につながった

1. はい
2. いいえ
3. わからない

(10) 将来的に役立つものだった

1. はい
2. いいえ
3. わからない

(11) 時間の節約につながった

1. はい
2. いいえ
3. わからない

---

引き続き、診療にあたって図書館情報資源を探したり使ったりした同じ時のことについてお答えください。

1.9 その情報によって診療上好ましい変化がありましたか？

1. はい
2. どちらかといえば「はい」
3. どちらかといえば「いいえ」
4. いいえ

[1.9 で「1.はい」「2. どちらかといえばはい」と回答した方は 1.9a へ、その他の方は 1.10 へお進みください]

1.9a どのような変化か、以下から選択してください(複数回答可)

1. 診断
2. 検査の選択
3. 薬剤の選択
4. 治療の選択
5. 入院期間の短縮
6. 退院後のケアや治療
7. 患者や家族への助言
8. その他（具体的に：　　　　　　　　　　　　　　　）

---

引き続き、診療にあたって図書館情報資源を探したり使ったりした同じ時のことについてお答えください。

1.10 その情報によって回避できたことはありますか？

1. はい
2. どちらかといえば「はい」
3. どちらかといえば「いいえ」
4. いいえ

[1.10 で「1.はい」「2. どちらかといえばはい」と回答した方は 1.10a へ、その他の方は 1.11 へお進みください]

1.10a どのようなことを回避できたのか、以下から選択してください(回答はいくつでも)

1. 入院
2. 再入院
3. 患者の死亡
4. 言語や文化の誤解
5. 疾患に関する患者の誤解
6. 院内感染
7. 外科手術
8. 規則や規程に対する違反
9. 過剰な検査や処置
10. 薬の副作用や相互作用

11. 医療過誤
12. 誤診
13. その他（具体的に：　　　　　　　　　　　　　　　　　）

---

引き続き、診療にあたって図書館情報資源を探したり使ったりした同じ時のことについてお答えください。

1.11 今回思い起こしていただいているケースで、図書館情報資源とその他の情報源について、それぞれ重要性を評価してください。その他に使った情報源があれば、(5)に入力の上、評価してください。

(1) 図書館情報資源
1. とても重要である
2. 重要である
3. あまり重要でない
4. まったく重要でない
5. 今回は使っていない

(2) 画像診断情報
1. とても重要である
2. 重要である
3. あまり重要でない
4. まったく重要でない
5. 今回は使っていない

(3) 臨床検査情報
1. とても重要である
2. 重要である
3. あまり重要でない
4. まったく重要でない
5. 今回は使っていない

(4) 同僚との意見交換
1. とても重要である
2. 重要である
3. あまり重要でない
4. まったく重要でない
5. 今回は使っていない

(5) その他（具体的に：　　　　　　　　　　　　　　　）
1. とても重要である
2. 重要である

3. あまり重要でない
4. まったく重要でない
5. 今回は使っていない

---

引き続き、診療にあたって図書館情報資源を探したり使ったりした同じ時のことについてお答えください。

1.12 あなたの診療上の疑問を解決するための情報ニーズについてコメントがあればお書きください。

---

セクション 2：あなたご自身についてお尋ねします。

調査結果の分析のために以下の質問にお答えください

2.1 あなたが取得された最高学位は？

1. 学士
2. 修士
3. 博士
4. その他（具体的に：　　　　　　　　　　　　　　）

2.2 あなたの年齢は？

1. 20 歳未満
2. 20～29 歳
3. 30～39 歳
4. 40～49 歳
5. 50～59 歳
6. 60～69 歳
7. 70 歳以上

2.3 あなたの性別は？

1. 男性
2. 女性

2.4 あなたの医療職としての経験年数は？

1. 2 年未満
2. 2 年以上 5 年未満
3. 5 年以上 10 年未満

4. 10 年以上 15 年未満
5. 15 年以上 20 年未満
6. 20 年以上

2.5 本調査についてお気づきのことがあれば、お書きください。

---

[終了画面]
ご協力ありがとうございました。質問は以上です。

あなたのご回答を、医療のためにより質の高い健康情報サービスに役立たせていただきます。お気づきの点がありましたら下記へご連絡ください。

連絡先：○○○（内線：　　　email：　　　）

完了

---

* This document shows the actual version of questionnaire for the current study in Japan. The questions were replicated and translated based on the appendix A "Facilitator Handbook Revised 2011" attached to the Value Study in the U.S. below:
Marshall JG, Sollenberger J, Easterby-Gannett S, Morgan LK, Klem ML, Cavanaugh SK, et al. The value of library and information services in patient care: results of a multisite study. J Med Libr Assoc. 2013;101(1): 38-46. doi: 10.3163/1536-5050.101.1.007
