# Supplementary material for: Clinical usefulness of library and information services in Japan: The detailed use and value of information in clinical settings
Source: PLoS One. 2018 Jun 28;13(6):e0199944. doi: 10.1371/journal.pone.0199944 (PMC6023225; doi:10.1371/journal.pone.0199944)
Supplement: S2 Appendix — (PDF) [file pone.0199944.s002.pdf]

S1_File The survey questionnaire in English*

Welcome to the Value Study!

Dear Physicians, Residents, and Nurses at the xxx hospital:

Your responses will help us to improve access to the library and information resources that you need for patient care. Please review the information about this survey in the request email and click the confirmation button below to begin.

[Radio Button] I was informed about this survey

Section1: We are asking your information needs to resolve questions in your clinical care

1.1. What is your profession?

1. Physician
2. Junior resident
3. Senior resident
4. Nurse
5. Nurse(Manager)
6. Other(Please specify :   )

1.1a. Which of the following does your job involve best?

1. Medical care
2. Nursing care
3. Medical and nursing care
4. Other (Please specify:)

1.1b Which of the following does your job involve (Select all that apply)

1. Clinical research
2. Basic research
3. Education
4. Management/Administration
5. Other (Please specify:)

[Respondents who select '4. Other' in Question 1.1b please go to 2.1]

Thinking of an occasion in the last 6 months when you looked for information resources other than electronic patient records and lab test results such as library served database and electronic journals for patient care, please answer the following questions

1.1c_1st What was the diagnosis of the patient (Select all that apply)
1.1c_2nd What was the principal diagnosis of the patient (Select only one answer)

1. Infectious diseases and parasitic diseases
2. Cancer
3. Diseases of the blood and blood-forming. Impairment of immune mechanism
4. Endocrine, nutritional and metabolic diseases
5. Disorders of mental and behavior
6. Diseases of the nervous system
7. Diseases of the eyes and appendages
8. Diseases of ear and mastoid process
9. Diseases of cardiovascular system
10. Disease of the respiration system
11. Diseases of the digestive system
12. Diseases of the skin and subcutaneous tissue
13. Musculoskeletal and connective tissue diseases
14. Disease of renal ureter reproductive system
15. Pregnancy, childbirth and postpartum
16. Conditions that occurred during perinatal period
17. Congenital malformations, deformations and chromosomal abnormalities
18. Damage, poisoning and other external disorders
19. Other (Please specify :)

Continuously thinking of the same occasion when you looked for information resources such as library served database and electronic journals for patient care, please answer the following questions.

1.2. What type of information did you need to answer the question? (Select all that apply)

1. Drug information
2. Therapy information
3. Clinical procedure
4. Diagnosis

5. Information for patient
6. Prognosis (Outcome)
7. Adverse effects
8. Patient safety
9. Clinical guidelines
10. Other (Please specify :   )

Continuously thinking of the same occasion when you looked for information resources such as library served database and electronic journals for patient care, please answer the following questions.

1.3 What resources did you use to search for the information you needed to answer your question? (Select all that apply)

English resources

1. Books (online)
2. Books (print)
3. Clinical Evidence (BMJ)
4. DynaMed
5. UpToDate
6. Cochrane Library
7. ClinicalKey(Elsevier)
8. PubMed
9. CINAHL
10. Electronic journals
11. Printed magazines
12. Web site of academic organizations
13. Other (Please specify:)

Japanese resources

14. Books (online)
15. Books (print)
16. Ichushi Web
17. JDream III
18. Current Index to Japanese Nursing Literature
19. Electronic journals
20. Printed magazines
21. Web site of academic organizations
22. Medical Information Network Distribution Service
23. Other (Please specify:)

24. Except for English and Japanese (Please specify:)
25. Not sure

Continuously thinking of the same occasion when you looked for information resources such as library served database and electronic journals for patient care, please answer the following questions.

1.4 How did you get to the resource(s) that you used?

1. In your institution's library
2. On your institution's library website
3. Asked your librarian or library staff
4. In other institution's library
5. On other institution's library website
6. Asked other librarian or library staff
7. Asked colleague
8. Personal/departmental subscription
9. Asked medical representative of pharmaceutical company
10. Search engine such as Goggle
11. Bookmarked website
12. Nothing
13. Other (Please specify:)
14. Not sure

1.5 From what physical location did you conduct your search for information?

1. In the library (the physical place)
2. Office
3. Home
4. Patient care unit
5. Other (Please specify:)

Continuously thinking of the same occasion when you looked for information resources such as library served database and electronic journals for patient care, please answer the following questions.

1.6 Did you find the information you needed to resolve the question in clinical care?

1. Completely
2. Partially
3. Not at all

[Respondents who selected ‘2. Partially’ or ‘3. Not al all’ in Question 1.6, please go to 1.6a. Other respondents please go to 1.7]

1.6a Why were you unable to find the information you needed?

1. Ran out of time
2. Information did not exist
3. Couldn’t find the information
4. Other (Please specify:)

[Respondents who selected ‘3. Not at all’ in Question 1.6, please skip questions 1.7-12]

Continuously thinking of the same occasion when you looked for information resources such as library served database and electronic journals for patient care, please answer the following questions.

1.7 Which resource(s) included relevant information?

English resources

1. Books (online)
2. Books (print)
3. Clinical Evidence (BMJ)
4. DynaMed
5. UpToDate
6. Cochrane Library
7. ClinicalKey(Elsevier)
8. PubMed
9. CINAHL
10. Electronic journals
11. Printed magazines
12. Web site of academic organizations
13. Other (Please specify:)

Japanese resources

14. Books (online)
15. Books (print)
16. Ichushi Web
17. JDream III
18. Current Index to Japanese Nursing Literature
19. Electronic journals

20. Printed magazines
21. Web site of academic organizations
22. Medical Information Network Distribution Service
23. Other (Please specify:)

24. Except for English and Japanese (Please specify:)
25. Not sure

Continuously thinking of the same occasion when you looked for information resources such as library served database and electronic journals for patient care, please answer the following questions.

1.7a As a result of the information you obtained, did you handle any aspect of the clinical situation differently than you would have handled it otherwise? (Select only one response)

1. Definitely yes
2. Probably yes
3. Probably no
4. Definitely no

1.8 Please indicate whether you agree or disagree with each of the following statements about the information you used:

(1) The information was relevant
1. Agree
2. Disagree
3. Do not know

(2) The information was accurate
1. Agree
2. Disagree
3. Do not know

(3) The information was current
1. Agree
2. Disagree
3. Do not know

(4) The information refreshed my memory of detail or facts
1. Agree
2. Disagree
3. Do not know

(5) The information substantiated my prior knowledge or belief

1. Agree
2. Disagree
3. Do not know

(6) The information provided new knowledge

1. Agree
2. Disagree
3. Do not know

(7) The information was of clinical value

1. Agree
2. Disagree
3. Do not know

(8) The information resulted in a better clinical decision

1. Agree
2. Disagree
3. Do not know

(9) The information contributed to higher quality of care

1. Agree
2. Disagree
3. Do not know

(10) The information will be of use in the future

1. Agree
2. Disagree
3. Do not know

(11) Having the information saved me time

1. Agree
2. Disagree
3. Do not know

Continuously thinking of the same occasion when you looked for information resources such as library served database and electronic journals for patient care, please answer the following questions.

1.9 Did any positive change(s) as a result of the information?

1. Definitely yes
2. Probably yes
3. Probably no
4. Definitely no

[Respondents who selected ‘1. Definitely yes’ or ‘Probably yes’ in Question 1.9, please goto 1.9a. Other respondents please go to 1.10]

1.9a Which of the following were the positive changes as a result of the information? (Select all that apply)

1. Diagnosis
2. Choice of test
3. Choice of drugs
4. Choice of treatment
5. Length of stay (reduced)
6. Post-hospital care or treatment
7. Changed advice given to patient
8. Other

Continuously thinking of the same occasion when you looked for information resources such as library served database and electronic journals for patient care, please answer the following questions.

1.10 Did any avoided unwelcome event(s) as a result of the information?

1. Definitely yes
2. Probably yes
3. Probably no
4. Definitely no

[Respondents who selected ‘1. Definitely yes’ or ‘Probably yes’ in Question 1.10, please go to 1.10a. Other respondents please go to 1.11]

1.10a Which of the following did you avoid as a result of the information? (Select all that apply)

1. Hospital admission
2. Hospital readmission
3. Patient death
4. Language/culture misunderstanding
5. Patient misunderstanding of disease
6. Hospital acquired infection
7. Surgery
8. Regulatory non-compliance
9. Additional tests or procedures
10. Adverse drug reaction or interaction

11. Medication error
12. Misdiagnosis
13. Other (Please specify:)

Continuously thinking of the same occasion when you looked for information resources such as library served database and electronic journals for patient care, please answer the following questions.

1.11 Please rate the importance of the information you received from the library and other sources in relation to this medical situation. Please indicate another information source you may have used and rate the source in (5).

(1) Library served information resource
1. Very important
2. Important
3. Not very important
4. Not at all important
5. Not use

(2) Diagnostic imaging
1. Very important
2. Important
3. Not very important
4. Not at all important
5. Not use

(3) Lab tests
1. Very important
2. Important
3. Not very important
4. Not at all important
5. Not use

(4) Discussion with colleagues
1. Very important
2. Important
3. Not very important
4. Not at all important
5. Not use

(5) Other (Please specify:)
1. Very important
2. Important

3. Not very important
4. Not at all important
5. Not use

Continuously thinking of the same occasion when you looked for information resources such as library served database and electronic journals for patient care, please answer the following questions.

1.12 Additional comments on your information needs.

Section2: We are asking questions about yourself.

Please answer the questions which will assist in survey analysis.

2.1 What academic degree did you earn as the highest?

1. Bachelor's
2. Master's
3. Doctorate
4. Other (Please specify:)

2.2 What is your age category?

1. Under 20 years
2. 20-29 years
3. 30-39 years
4. 40-49 years
5. 50-59 years
6. 60-69 years
7. Over 70 years

2.3 What is your gender?

1. Men
2. Women

2.4 How many years have you been working as a health professional?

1. Less than 2 years
2. 2 years - less than 5 years
3. 5 years-less than 10 years

4. 10 years-less than 15 years
5. 15 years-less than 20 years
6. More than 20 years

2.5 Please add any additional comments below.

END SCREEN

Thank you for your cooperation.

Your responses will be analyzed to deliver high quality information services for clinical care. For any questions and concerns please contact below:

Name xxxxx (ext. xxxxx email.xxxxx)

---

* The survey was conducted in Japanese. This document shows the reverse translation into English. The questions were replicated based on the appendix A "Facilitator Handbook Revised 2011" attached to the Value Study in the U.S. below:
Marshall JG, Sollenberger J, Easterby-Gannett S, Morgan LK, Klem ML, Cavanaugh SK, et al. The value of library and information services in patient care: results of a multisite study. J Med Libr Assoc. 2013;101(1): 38-46. doi: 10.3163/1536-5050.101.1.007
